# Agreement and Diagnostic Performance of Urinary HPV Testing Versus Clinician-Collected Cervico-Vaginal Sampling: A Prospective Cross-Sectional Study in a Romanian Cohort

**DOI:** 10.3390/medsci14040423

**Published:** 2026-07-24

**Authors:** Ionel-Daniel Nati, Mihaela Oancea, Carmen Mihaela Mihu, Dan Mihu, Razvan Ciortea, Cristian Iuhas, Carmen Bucuri, Maria Patricia Roman, Cristina Mihaela Ormindean, Viorela Suciu, Razvan Chereches, Andrei Mihai Malutan

**Affiliations:** 1 2nd Department of Obstetric and Ginecology, “Iuliu Hatieganu” University of Medicine and Pharmacy, 400610 Cluj-Napoca, Romania; ionel.nati@umfcluj.ro (I.-D.N.);; 2 Department of Morphofunctional Sciences, Iuliu Hațieganu University of Medicine and Pharmacy, 400012 Cluj-Napoca, Romania; 3 HIVE Health Innovation Center, Babeș-Bolyai University, 400094 Cluj-Napoca, Romania

**Keywords:** human papillomavirus, urine test, urine HPV, cervical cancer screening, CIN2+, self-sampling, Romania

## Abstract

**Background:** Cervical cancer screening uptake in Romania remains below 20%, with invasive sampling cited as a major participation barrier. Urinary HPV testing is a non-invasive alternative, but data from Eastern European populations are scarce and reporting stratified by histological grade is inconsistent. **Objectives:** To evaluate the agreement between paired urinary and clinician-collected cervico-vaginal HPV testing, and to assess the diagnostic performance of urinary HPV testing for biopsy-confirmed cervical intraepithelial neoplasia grade 2 or worse (CIN2+). **Methods:** Prospective cross-sectional study in three outpatient obstetrics and gynecology clinics (May 2025–May 2026). A total of 230 women aged 25–70 years provided paired cervico-vaginal (PreservCyt^®^) and first-void urine (Colli-Pee^®^) specimens. To capture the full spectrum of disease probability, participants were recruited across three clinical scenarios: primary screening, cytology triage and HPV-positive triage. A multiplex RT-PCR assay targeted a total of 14 high-risk HPV genotypes, providing separate identification for HPV16 and HPV18, alongside a pooled detection for the remaining 12 high-risk strains. Colposcopy-guided cervical biopsy served as the reference standard. **Results:** Paired hrHPV testing achieved substantial agreement (κ = 0.696, 95% CI 0.604–0.788), with higher genotype-specific concordance for HPV16 (κ = 0.755) and HPV18 (κ = 0.789). Discordance was directionally asymmetric (30 cervical-positive/urine-negative versus 4 urine-positive/cervical-negative; McNemar *p* < 0.001). For biopsy-confirmed CIN2+ (59 of 230; 25.7%), urinary hrHPV showed a sensitivity of 86.4% (95% CI 75.5–93.0), specificity 56.7%, positive predictive value 40.8% and negative predictive value 92.4%, compared with 91.5%, 43.3%, 35.8% and 93.7% for cervico-vaginal testing. Urinary HPV positivity increased monotonically with histological severity (25.0% no-lesion, 69.0% CIN1, 86.4% CIN2+; linear-by-linear *p* < 0.001). **Conclusions:** Urinary HPV testing demonstrates substantial agreement with clinician-collected sampling, near-equivalent negative predictive value, and a robust dose–response with histological severity. It is a clinically credible non-invasive entry point for a triage cascade in settings such as Romania, where low participation rather than analytical performance is the principal screening barrier.

## 1. Introduction

Cervical cancer remains one of the leading causes of oncological morbidity and mortality among women worldwide, with a disproportionate burden in low- and middle-income countries [1,2]. The etiology is, in the vast majority of cases, driven by persistent infection with high-risk oncogenic human papillomavirus types (hrHPV) [3], and the identification of this causal link has fundamentally reshaped the screening paradigm: molecular hrHPV testing has become the reference instrument for prevention programs, outperforming conventional cytology in sensitivity for the detection of high-grade lesions (CIN2+) [1,4].

Nevertheless, effective participation in screening remains suboptimal. Cultural, logistical, and personal-comfort barriers—particularly reluctance toward invasive gynecological examination—exclude from prevention precisely the population segments at highest risk [1,5,6]. In this context, HPV testing on self-collected specimens has emerged as a strategic alternative, with urine showing the highest acceptability profile: a fully non-invasive sample, easily obtained at home, and compatible with large-scale screening programs [1,5,7]. Urinary HPV testing has been defined as a non-invasive nucleic acid amplification method for detecting oncogenic HPV DNA in urine specimens, as an alternative to cervical sampling [1,7].

The biological basis for this approach is now well documented: urine specimens contain HPV DNA originating from exfoliated cervical cells, allowing infection to be detected via sensitive PCR methods [8,9,10]. The meta-analysis by Miazga et al. reports a pooled sensitivity of 87% (95% CI: 0.74–0.94) and specificity of 89% (95% CI: 0.81–0.93) for HPV detection in first-void urine (FVU) [8], while Cho et al. show that, despite a systematic reduction in urinary sensitivity compared to clinician-collected samples (relative ratio = 0.84), modern PCR assays approach near-complete equivalence (relative sensitivity = 0.98) [11]. Concordance between genotypes detected in urine and those identified in cervical samples is consistently highest for HPV16 [12,13]—the genotype contributing most heavily to invasive cancer cases [14].

Despite this growing evidence base, the literature presents several gaps that limit the translation of urinary HPV testing into routine screening practice. First, a unified standard for the pre-analytical protocol is lacking: there are no established recommendations on the type of urine collected (FVU vs. midstream), optimal volume, DNA preservation medium, or extraction method [1,9,10,15]. This methodological heterogeneity likely explains the wide variability in reported sensitivities [1,8,16]. Second, the majority of diagnostic accuracy studies have been conducted in clinically selected populations—women referred to colposcopy for abnormal cytology—which introduces spectrum bias [17,18]. Third, performance reporting stratified by histological grade (no intraepithelial lesion/CIN1/CIN2+) remains inconsistent, even though such stratification is essential to determine whether the urinary test can discriminate clinically significant lesions from indolent ones or from the absence of disease. In addition, data from Eastern European populations remain scarce, and local epidemiological particularities may meaningfully influence the actual diagnostic value of the method [19].

Building on these gaps, the present study addresses whether urinary HPV testing can serve as a clinically meaningful alternative to clinician-collected cervico-vaginal sampling in a population that spans the full histological spectrum of cervical disease. Previous reports have shown that urinary and cervical HPV results yield substantial concordance (κ in the range of 0.55–0.75) [12,13], and that HPV16 displays the most consistent reproducibility across biological matrices [12]. What remains insufficiently established is whether urinary positivity tracks the histological severity of the underlying lesion in a manner robust enough to support its integration into screening pathways, and whether this relationship holds once classical risk factors such as smoking and age are taken into account. To clarify this question, the present work jointly examines cervico-vaginal HPV testing, urinary HPV testing, and colposcopic biopsy, with the broader aim of strengthening the evidence base required for incorporating urinary HPV testing into cervical cancer screening, particularly for population segments in which conventional clinical sampling remains a substantive access barrier.

## 2. Materials and Methods

### 2.1. Study Design

We conducted a prospective, observational, cross-sectional study between May 2025 and May 2026 in three outpatient obstetrics and gynecology clinics located within a single region of north-western Romania (the Cluj–Bistrița-Năsăud area), serving a shared and partly overlapping catchment population and operating under a common clinical protocol, with referral to a single laboratory and a single pathology service. The multi-clinic structure was adopted for methodological rather than volumetric reasons: to avoid single-operator and single-site sampling bias, to broaden the referral spectrum across the three clinical entry scenarios, and to verify that the pre-analytical protocol was reproducible across independent clinical teams. This study should therefore be understood as a single-region study conducted across three collaborating clinics, not as a national multicenter consortium. The primary objective was to evaluate the agreement between HPV testing performed on urine samples and HPV testing performed on cervico-vaginal samples collected by the gynecologist, using Cohen’s kappa coefficient. The secondary endpoint addressed the diagnostic performance of urinary HPV testing for the identification of CIN2+ cervical lesions, with histopathological examination serving as the reference standard, in line with the STARD 2015 (Standards for Reporting of Diagnostic Accuracy Studies) recommendations. The sample size was determined a priori to ensure adequate statistical power for both the primary agreement analysis and the secondary diagnostic accuracy endpoints. Based on contemporary meta-analytical data estimating the pooled sensitivity of urinary HPV testing at approximately 85%, a minimum of 49 true-positive (CIN2+) cases was required to estimate sensitivity with a precision margin of ±10% at a 95% confidence level. Assuming a conservative CIN2+ prevalence of 20% to 25% in a mixed outpatient setting of primary screening and cytology triage, a total sample size of 200 to 250 women was targeted. Furthermore, this target exceeds the approximately 150 paired samples required to provide 80% power to detect a substantial agreement (Cohen’s κ ≥ 0.70) against a null hypothesis of moderate agreement (κ = 0.50) with a two-sided α of 0.05.

The study was conducted in accordance with the principles of the Declaration of Helsinki and was approved by the Ethics Committee of the “Iuliu Haţieganu” University of Medicine and Pharmacy, Cluj-Napoca, Romania (IH903419 from 18 February 2025). All participants provided written informed consent prior to enrollment. Data were anonymized before statistical analysis.

### 2.2. Patient Recruitment and Eligibility Criteria

To ensure the study population reflected the full spectrum of cervical disease probability encountered in routine practice, participants were recruited across three concurrent clinical scenarios:

- Primary screening: Asymptomatic women attending for routine gynecological check-ups with no known cervical pathology;
- HPV-positive triage: Patients with positive cervico-vaginal HPV screening test attending for follow-up cytology;
- Cytology triage: Patients with abnormal cervical cytology result presenting for further evaluation or colposcopy.

For all participants, regardless of clinical entry point, both specimens were obtained during a single clinical visit, following a fixed collection sequence in which the first-void urine specimen was always collected first.

Each participant was enrolled once and only once, at a single clinical entry point, and contributed exactly one paired specimen set. The enrolment register was deduplicated across the three participating clinics prior to any analysis; a woman already enrolled who subsequently returned for follow-up under a different scenario was not re-enrolled, and no further specimens were collected. The scenario recorded for each participant is therefore the one prevailing at the single moment of enrolment, and no individual is counted in more than one stratum.

Exclusion criteria were: refusal to provide any of the biological samples; inability to provide informed consent; age below 25 years or above 70 years; current pregnancy; prior hysterectomy; and active treatment for cervical dysplasia at the time of enrolment. Prior HPV vaccination was not an exclusion criterion, and vaccination status was not recorded as a study variable; the rationale and the implications of this choice are addressed in the Discussion and Limitations.

### 2.3. Collection and Processing of Biological Samples

A fixed collection sequence was applied to all participants, with the first-void urine specimen invariably obtained first. Participants voided into the Colli-Pee device (Novosanis NV, Wijnegem, Belgium) in the clinic restroom immediately upon arrival, before any speculum insertion, before any contact of the genital tract with lubricant or antiseptic, and before any cervical manipulation, cleansing, mucus removal or brushing. The cervico-vaginal brushing and, where clinically indicated, colposcopy with acetic acid and Lugol’s iodine and biopsy were performed subsequently within the same visit. This sequence was adopted deliberately: because first-void urine acquires its diagnostic content through the passive washing of exfoliated cervical and vaginal cells, mucus and cellular debris into the initial urinary stream, any prior procedure that depletes this cellular and mucous load, or that introduces interfering substances into the lower genital tract, could plausibly degrade the urinary HPV signal. Collecting the urine specimen first protects the matrix from such pre-analytical depletion and from lubricant-mediated interference.

The cervico-vaginal specimen was collected by the gynecologist using a dedicated cervical brush and placed in PreservCyt^®^ liquid-based medium (ThinPrep^®^, Hologic Inc., Marlborough, MA, USA), in keeping with standard protocols for liquid-based cytology. Samples were transported and processed under standardized conditions for viral DNA extraction and HPV genotyping.

Urine samples were collected using the Colli-Pee^®^ device (Novosanis NV, Wijnegem, Belgium), which standardizes collection of the first-void urine fraction. Neither first-morning nor fasting collection was required. Samples were stored at 4 °C until processing. For DNA extraction, a 1 mL aliquot of the preserved first-void urine was centrifuged at 7500 rpm for 10 min; after removal of the supernatant, the pellet was resuspended in 200 µL of phosphate-buffered saline (PBS).

### 2.4. HPV Detection and Genotyping

Viral DNA extraction was carried out with extraction kits from Tianlong Science & Technology Co., Ltd. (Xi’an, China) on the automated GeneRotex96 platform, strictly following the manufacturer’s instructions. The dedicated HPV nucleic acid extraction kit was used for cervico-vaginal samples, while the viral DNA/RNA extraction kit was used for urine samples after the prior centrifugation and resuspension step. HPV detection and genotyping were performed using the commercially available Tianlong Human Papillomavirus (HPV) 14 High-Risk Types Nucleic Acid Detection Kit (Tianlong Science & Technology Co., Ltd., Xi’an, China). This automated in vitro real-time PCR (RT-PCR) and molecular hybridization assay targets an approximately 200-nucleotide sequence within the L1 polymorphic region of the HPV genome. A multiplex RT-PCR assay targeted a total of 14 high-risk HPV genotypes, providing separate identification for HPV16 and HPV18, alongside a pooled detection for the remaining 12 high-risk strains. The system incorporates a beta-globin internal control to monitor sample adequacy, alongside standard positive and negative analytical controls.

For each participant, we recorded clinical and demographic data covering age, smoking status, educational level, the cervico-vaginal cytology result according to the Bethesda 2014 classification, the cervical biopsy result, and, when available, the histopathological examination of the conization specimen. Histopathological results were classified according to the CIN system as: no lesion, CIN1, CIN2, CIN3, carcinoma in situ, and invasive carcinoma.

To minimize bias in accordance with STARD 2015 guidelines, strict blinding was applied to the investigational analytical pathways. Laboratory technicians performing the multiplex RT-PCR assays were fully blinded to the participants’ clinical data, cytological findings, and histological diagnoses. Clinical personnel collected the paired samples within a single visit following the fixed urine-first sequence described in Section 2.3, which was adopted specifically to protect the first-void urine matrix from pre-analytical depletion and from interference by lubricants or antiseptics. We note that concurrent collection does not, of itself, eliminate collection-sequence bias; the fixed sequence, rather than concurrency, is what standardizes the pre-analytical conditions across participants. However, to strictly adhere to the informed consent provisions stating that study participation would not alter standard-of-care management, the pathologists evaluating the cervical biopsies were not blinded to the routine clinical referral data (including prior cytology and cervico-vaginal HPV status). Crucially, the pathologists remained completely blinded to the investigational urinary HPV test results.

### 2.5. Statistical Analysis

Statistical analysis was performed in SPSS version 23.0 (IBM Corp., Armonk, NY, USA). The threshold for statistical significance was set at α = 0.05, two-sided, with 95% confidence intervals.

Continuous variables were summarized as mean and standard deviation or as median and interquartile range, depending on their distribution. Categorical variables were expressed as absolute frequencies and percentages. Normality of distribution was assessed with the Shapiro–Wilk and Kolmogorov–Smirnov tests.

Agreement between the two HPV testing methods was evaluated using Cohen’s kappa coefficient with 95% confidence intervals. For multi-category variables, a quadratic-weighted kappa was used. Kappa values were interpreted according to the classification of Landis and Koch. The symmetry of discordances was assessed with McNemar’s test applying Yates’ continuity correction.

For the analysis of diagnostic performance, a composite reference standard was applied: histological diagnosis from cervical biopsy or conization specimens was used for the 140 women who underwent the procedure, while the absence of disease was clinically inferred for the 90 unbiopsied women lacking any indication for colposcopy. Sensitivity, specificity, positive predictive value, and negative predictive value were calculated, with 95% confidence intervals derived using the Wilson method.

Although identifying clinical predictors of discordance (such as age or cytological grade) is of theoretical interest, the limited number of discordant events in our cohort (*n* = 34), predominantly driven by compartmental dilution, precluded a robust multivariable analysis without the risk of overfitting.

The reporting of the diagnostic accuracy component followed the STARD 2015 recommendations, and the agreement component was framed in accordance with the methodological principles for reliability and agreement studies (GRRAS).

## 3. Results

### 3.1. Baseline Characteristics of the Study Population

A total of 230 women were enrolled and contributed paired cervico-vaginal and urinary HPV specimens between May 2025 and May 2026. Following the clinical context at presentation, the study population encompassed three distinct clinical scenarios: 98 women (42.6%) presented for abnormal cytology triage, 86 women (37.4%) attended for follow-up cytology after a prior positive cervico-vaginal hrHPV screening test, and 46 asymptomatic women (20.0%) were recruited during routine primary screening. Participants had a mean age of 38.9 years and 87 (37.8%) were active smokers.

Clinical indication for colposcopy-guided biopsy was met in 140 of the 230 enrolled women (60.9%). For the remaining 90 participants—who presented with negative screening results and lacked clinical indication for invasive procedures—the absence of high-grade disease was inferred via standard clinical triage algorithms. Based on this composite reference standard, high-grade disease (CIN2+) was histopathologically confirmed in 59 women (25.7%); CIN1 was present in 71 women, while 100 women (10 biopsy-confirmed and 90 clinically inferred) were classified as having no intraepithelial lesion.

Overall hrHPV positivity was 65.7% on clinician-collected cervico-vaginal samples and 54.3% on urine. Full demographic, cytological, histological and virological characteristics are provided in Table 1.

### 3.2. Agreement Between Urinary and Cervico-Vaginal HPV Testing

For pooled high-risk HPV detection, paired results were concordant in 196 of 230 participants (overall raw agreement 85.2%), with 121 dual-positive and 75 dual-negative pairs. Discordance was directionally asymmetric: 30 participants (13.0%) were cervico-vaginally HPV-positive but urine-negative, whereas only 4 (1.7%) were urine-positive but cervico-vaginally HPV-negative. Cohen’s kappa for any-hrHPV detection reached 0.696 (95% CI 0.604–0.788; *p* < 0.001), corresponding to substantial agreement on the Landis–Koch scale. McNemar’s test with continuity correction confirmed a statistically significant directional difference in marginal positivity rates between the two matrices (χ^2^ = 18.38, *p* < 0.001), indicating systematically higher positivity in the cervico-vaginal compartment.

A complementary diagnostic-test framing of the same paired data, using the clinician-collected cervico-vaginal result as the operative reference, quantifies the directional asymmetry more transparently. Under this framing, urinary hrHPV achieved a sensitivity of 80.1% (95% CI 73.0–86.0) and a specificity of 94.9% (95% CI 88.0–99.0) relative to the cervico-vaginal compartment, with a positive predictive value of 96.8% (95% CI 92.0–99.0) and a negative predictive value of 71.4% (95% CI 62.0–80.0). The same metrics for genotype-specific detection were 76.8%, 96.0%, 86.0% and 92.8% for HPV16 and 76.9%, 99.1%, 83.3% and 98.6% for HPV18 (sensitivity, specificity, positive predictive value and negative predictive value, respectively). The very high positive predictive value indicates that a urine-positive result was, in practice, virtually always accompanied by cervico-vaginal positivity, whereas the comparatively lower NPV reflects the same compartmental dilution evidenced by the asymmetric discordance described above.

Agreement was higher for genotype-specific analyses. For HPV16, concordance was observed in 210 of 230 pairs (91.3%), with 43 dual-positive and 167 dual-negative results; discordant pairs were balanced (13 cerv+/urine− and 7 cerv−/urine+), yielding Cohen’s κ = 0.755 (95% CI 0.653–0.857; *p* < 0.001) and a non-significant McNemar test (χ^2^ = 1.25, *p* = 0.264). For HPV18, 225 of 230 pairs were concordant (97.8%; 10 dual-positive, 215 dual-negative); 3 cases were cerv+/urine− and 2 cerv−/urine+, yielding Cohen’s κ = 0.789 (95% CI 0.609–0.969; *p* < 0.001) without evidence of directional bias (exact McNemar *p* = 1.000). Pooled and genotype-specific concordance metrics are summarized in Table 2.

### 3.3. Formatting of Mathematical Components

Considering colposcopy-guided biopsy as the reference standard, 59 of 230 women (25.7%) had biopsy-confirmed CIN2+. Urinary hrHPV testing correctly identified 51 of these lesions, yielding a sensitivity of 86.4% (95% CI 75.5–93.0) and a specificity of 56.7% (95% CI 49.2–63.9); the corresponding positive predictive value was 40.8% (95% CI 32.6–49.6) and the negative predictive value 92.4% (95% CI 85.7–96.1). The crude odds ratio for CIN2+ in urine-positive versus urine-negative women was 8.36 (95% CI 3.74–18.68; Pearson χ^2^ = 32.94, *p* < 0.001).

In the same cohort, cervico-vaginal hrHPV testing achieved a sensitivity of 91.5% (95% CI 81.6–96.3), specificity of 43.3% (95% CI 36.1–50.8), PPV of 35.8% (95% CI 28.6–43.7) and NPV of 93.7% (95% CI 86.0–97.3) for CIN2+, with an odds ratio of 8.24 (95% CI 3.14–21.62; *p* < 0.001). Restricting positivity to women in whom both compartments were concurrently positive (*n* = 121) yielded a sensitivity of 81.4% (95% CI 69.6–89.3), a specificity of 57.3% (95% CI 49.8–64.5), a PPV of 39.7% (95% CI 31.4–48.6) and an NPV of 89.9% (95% CI 82.8–94.3), with an odds ratio of 5.86 (95% CI 2.85–12.06; *p* < 0.001). Diagnostic performance metrics for the three case definitions are juxtaposed in Table 3.

### 3.4. HPV Positivity Stratified by Histological Grade

Urinary hrHPV positivity increased monotonically with histological severity, from 25.0% (25/100) in women with no intraepithelial lesion, to 69.0% (49/71) in CIN1 and 86.4% (51/59) in CIN2+ (Pearson χ^2^ = 65.36, df = 2, *p* < 0.001; linear-by-linear association χ^2^ = 61.67, *p* < 0.001). A parallel gradient was observed for cervico-vaginal hrHPV positivity (34.0%, 88.7% and 91.5% across the same strata; χ^2^ = 78.72, df = 2, *p* < 0.001). The genotype-specific gradient was even more pronounced for HPV16: urinary HPV16 positivity rose from 9.0% in the no-lesion stratum to 18.3% in CIN1 and 47.5% in CIN2+ (χ^2^ = 32.97, df = 2, *p* < 0.001; linear-by-linear χ^2^ = 30.06, *p* < 0.001), while cervico-vaginal HPV16 positivity increased from 13.0% to 26.8% and 40.7%, respectively (χ^2^ = 15.76, df = 2, *p* < 0.001). Stratified positivity rates are reported in Table 4.

### 3.5. Association of Urinary HPV Testing with Cervical Cytology

Among all 230 participants, urinary hrHPV positivity rose from 23.7% (18/76) in NILM smears, to 65.8% (73/111) in ASC-US/LSIL and 74.4% (32/43) in ASC-H/HSIL specimens (Pearson χ^2^ = 44.17, df = 2, *p* < 0.001; linear-by-linear association χ^2^ = 38.48, *p* < 0.001), again indicating a graded, monotonic relationship between cytological abnormality and urinary HPV detection.

### 3.6. Additional Clinical Correlates of High-Grade Disease

The prevalence of active smoking increased with histological grade, from 24.0% in women without intraepithelial lesion, to 36.6% in CIN1 and 62.7% in CIN2+ (Pearson χ^2^ = 23.71, df = 2, *p* < 0.001; linear-by-linear association χ^2^ = 22.68, *p* < 0.001). Age differed across the three histological strata (Kruskal–Wallis H = 11.10, *p* = 0.004), with women in the CIN2+ group being younger (mean 35.1 ± 8.2 years; median 33, IQR 29–38) than those with no lesion (mean 41.1 ± 11.5 years; median 39, IQR 30–50). A pairwise Mann–Whitney comparison restricted to the no-lesion and CIN1 strata did not reach significance (U = 3234.5; Z = −0.99; *p* = 0.322), consistent with the bulk of the age gradient being driven by the high-grade stratum. The non-parametric Area Under the Curve (AUC) of age was 0.339 for the prediction of cervico-vaginal hrHPV positivity and 0.313 for urinary hrHPV positivity, both reflecting an inverse association between age and HPV detection rather than a discriminative diagnostic signal.

## 4. Discussion

In our cohort, which effectively captured the full histological spectrum of cervical disease, three clinically meaningful signals emerged. First, urinary and cervico-vaginal testing demonstrated substantial overall agreement, which predictably peaked for the highly oncogenic HPV16 and HPV18 genotypes. Second, while discordance was directionally asymmetric—favoring the clinician-collected swab in absolute detection rates—this finding was expected and reflects physiological compartmental dilution rather than an assay failure. Third, and most consequentially, urinary testing reliably identified the vast majority of high-grade (CIN2+) lesions. By matching the negative predictive value of conventional sampling while simultaneously outperforming it in specificity, the urinary matrix proves its viability as a robust, non-invasive diagnostic tool.

Taken together, these results support that even though urinary HPV testing in our cohort does not match the sensitivity of clinician-collected hrHPV testing for CIN2+, the shortfall is small in absolute terms (−5.1 percentage points) and is offset by a meaningful gain in specificity and a near-equivalent NPV. In a screening context where the principal aim is to safely rule out high-grade disease in a large population that is currently unscreened, the very high NPV is arguably the most decisive operating characteristic.

From a practical clinical perspective, the metrics presented in Table 3 translate into a highly efficient triage dynamic. While the cervico-vaginal test remains the gold standard for identifying true positives, its lower specificity (43.3%) invariably generates a higher volume of false alarms, potentially leading to unnecessary colposcopies. Conversely, urinary testing acts as a more stringent biological filter. For the clinician, a negative urinary HPV result is associated with a cross-sectional negative predictive value of 92.4% for high-grade disease (CIN2+), numerically close to the 93.7% obtained with clinician-collected sampling. This numerical proximity must not, however, be read as an equivalence of clinical reassurance, for the reasons developed below. Furthermore, its superior specificity (56.7%) and positive predictive value (40.8%) signify that a positive urine result is more tightly coupled to actual high-grade lesions, thereby reducing the clinical burden of over-investigating transient, low-risk infections.

Our overall κ of 0.696 falls within the substantial-agreement range described in the recent meta-analytic synthesis by Park et al., who quantitatively reviewed agreement statistics across self- versus clinician-collected samples and underscored the methodological limits of unweighted kappa in populations with skewed prevalence [31]. Our values are slightly lower than the 0.87 reported by Nilyanimit et al. on Colli-Pee-collected first-stream urine tested with the cobas 4800 system in 240 Thai women (96.7% concordance, 89% sensitivity and 98% specificity vs. cervical samples) [32], but very similar to the κ of 0.69 reported by Kim et al. in 210 South Korean asymptomatic women using a PANA RealTyper™ kit, who also found their urine-cervical concordance to be 93.3% [33]. Kappa is known to be deflated in low-prevalence settings and inflated when prevalence is moderate-to-high, so the convergence of our results with both extremes argues against the interpretation that urinary HPV is a “weaker” assay; instead, it indicates that the matrix continues to behave consistently across very different clinical populations.

Our genotype-specific κ values (0.755 for HPV16 and 0.789 for HPV18) align closely with the Chinese study by Song and Wang, who, in 458 women with cytological abnormalities, reported κ of 0.791 for HPV16 and 0.762 for HPV18 between urine and cervical cytology [34]. The convergence is striking given the methodological differences between the two cohorts, and supports a now well-replicated observation: paired-sample agreement increases as the genotype contributes more directly to high-grade disease, with HPV16 and HPV18—the two genotypes responsible for approximately 77% of invasive cervical cancer worldwide [35]—being the most reproducible across biological matrices. The recent United States cross-sectional study by Young et al. extended this further, reporting near-perfect prevalence-adjusted bias-adjusted kappa values (PABAK = 0.95) for HPV16 detection between first-void urine and clinician-collected speculum samples in 188 women [36]. Our point estimate sits between the lower bound of the Young cohort and the center of mass of the Korean/Chinese studies, which is the expected outcome for a mid-sized European triage cohort.

The genotype resolution afforded by our assay merits explicit contextualization. The Tianlong HPV 14 High-Risk Types kit resolves HPV16 and HPV18 individually and reports the remaining twelve high-risk genotypes as a single pooled channel. This configuration is not an idiosyncratic analytical choice but mirrors the prevailing national standard of care: the guideline of the Romanian Society of Obstetrics and Gynecology (SOGR) endorses molecular testing for the fourteen high-risk HPV genotypes, with mandatory separate reporting of HPV16 and HPV18—which carry a distinct, higher immediate risk of CIN3+ and therefore trigger direct referral to colposcopy—and pooled reporting of the remaining twelve types, which are triaged by reflex cytology [37]. The genotype information we generate is therefore precisely the information a Romanian clinician obtains in routine practice, which strengthens the direct programmatic transferability of our findings. Nevertheless, the pooled channel constrains the granularity of our concordance analysis. Extended genotyping platforms, such as the Onclarity assay, resolve HPV31, 33/58, 45, 51, 52, 56/59/66 and 35/39/68 as separate channels and permit finer risk stratification [38]; this is increasingly relevant, since the epidemiological weight of individual genotypes is shifting, with HPV18 no longer among the dominant types in several Central and Eastern European cohorts, while HPV31, 33, 45, 52 and 58 emerge as major contributors to high-grade disease [38].

The vaccination status of the cohort merits consideration, as it conditions the interpretation of genotype-specific findings. Romania has recorded among the lowest HPV vaccination coverage rates in the European Union, and a cohort of women aged 25–70 years enrolled in 2025–2026 (median age 36) is therefore drawn predominantly from birth cohorts in which vaccination uptake was negligible; the population may accordingly be regarded as essentially vaccine-naïve [19]. While this reflects a well-documented public health shortcoming, it confers a methodological advantage in the present context, minimizing the genotype-replacement effects that would confound concordance analyses in high-coverage populations. It should nevertheless be emphasized that vaccination does not obviate the need for screening: even the nonavalent formulation does not encompass the full spectrum of oncogenic genotypes, and current SOGR recommendations maintain unchanged screening intervals for vaccinated women [37]. Vaccination modifies the pre-test probability of infection and the distribution of genotypes, but it does not remove a woman from the screening population.

Our urinary HPV sensitivity of 86.4% for CIN2+ is consistent with the upper range of estimates in the contemporary literature. The umbrella review by Miazga et al. [8] pooled FVU sensitivity at 87% and specificity at 89%, while the meta-analysis of Cho et al. [11] documented near-equivalent relative sensitivity (0.98) for FVU. The randomized study by Davies et al. in 313 women referred to colposcopy in Manchester reported urine sensitivity for CIN2+ of 84% with the Colli-Pee device and confirmed superiority of FVU-device collection over standard pot collection [28], a finding that supports our methodological choice. In the same direction, the OncoPredict HPV Screening assay evaluated within the European VALHUDES framework by Giubbi et al. in 500 women referred to colposcopy demonstrated a CIN2+ sensitivity ratio of 0.95 (95% CI 0.88–1.02) for FVU versus matched cervical samples [39], and the Danish diagnostic test accuracy study by Tranberg et al. reached comparable conclusions in 2025 [20]. Our relative sensitivity of 0.94 (86.4%/91.5%) is therefore squarely within the consensus of recent European validation studies.

What is more distinctive in our data is the gradient with histological severity. Urinary hrHPV positivity rises monotonically from 25.0% in women with no lesion, to 69.0% in CIN1 and 86.4% in CIN2+, with parallel—and statistically robust—increases in cytological severity. This dose–response signal is conceptually different from binary sensitivity/specificity reporting and addresses the third major gap identified: whether urinary positivity tracks the clinically meaningful spectrum of disease. The linear-by-linear association (χ^2^ = 61.67, *p* < 0.001) confirms that it does. A similar gradient was reported in the umbrella review and meta-analytic literature [8,11], and in the United States MHOC cohort analysis by Young et al. [36], but few studies have presented the gradient with the granularity afforded by a population-level histological stratification, in particular for HPV16, where our urinary positivity rises from 9.0% in the no-lesion stratum to 47.5% in CIN2+.

The directional asymmetry of our discordance (30 cervical-positive/urine-negative versus only 4 urine-positive/cervical-negative pairs) deserves particular comment. This pattern is reproducible across the urinary HPV literature: Cho et al. previously reported a relative ratio of 0.84 for raw urinary sensitivity that improved to 0.98 only when modern PCR assays and FVU collection were combined [11]; Davies et al. observed the same direction with two devices [40]; and the foundational systematic review and meta-analysis by Pathak et al. explicitly documented this inherent dilution effect, confirming that urinary HPV detection systematically yields a lower raw sensitivity compared to direct cervical sampling [21]. The likely explanation lies upstream of the analytical assay: cellularity and viral load are lower in urine than in cervical brushings, because urinary HPV DNA originates from exfoliated cervical cells that reach the FVU stream through vaginal washing rather than directly from the lesion. Thirty cervical-positive/urine-negative discordances over 230 paired observations is therefore not a refutation of the urinary matrix; it is the expected consequence of compartmental dilution, and it is what specifically motivates the use of FVU devices with preservative. Crucially, a post hoc profiling of these 30 cervical-positive/urine-negative discordant cases revealed that the majority of ‘missed’ infections were of limited immediate clinical consequence: 80% (*n* = 24) harbored either no histopathological lesion (*n* = 9) or simple CIN1 (*n* = 15), whereas only 6 cases involved a CIN2+ lesion. This suggests that the physiological dilution inherent to the urinary matrix may inadvertently act as a biological filter, preferentially missing transient or low-viral-load infections while retaining a robust signal for clinically actionable, high-grade disease.

A critical caveat follows from this compartmental dilution: a negative urinary HPV result must not be equated with a negative clinician-collected result when determining the screening interval. The near-equivalent cross-sectional negative predictive values (92.4% versus 93.7%) quantify a different property from the longitudinal safety of an extended interval. A urine-negative result may reflect either the true absence of virus or a failure of detection for technical reasons: six of our thirty cervical-positive/urine-negative participants harbored a CIN2+ lesion. Until longitudinal data become available, a shortened re-testing interval for urine-negative women would be prudent.

The Romanian-specific implications of our findings deserve emphasis. As recently summarized by Saveliev et al., Romania continues to report the highest cervical cancer incidence and mortality in the European Union, with cervical screening uptake estimated to remain below 20% [19]. The contemporaneous SCCUT multicenter study, which enrolled 36,813 women from the South-Muntenia region between 2022 and 2023, found an overall hrHPV prevalence of 12.5% in a primary-screening population, with HPV16 attributable to a substantial fraction of the burden [22]. Our cohort, although clinically enriched relative to SCCUT, sits within the same epidemiological context: a population in which an acceptable, non-invasive screening modality could meaningfully shift the proportion of women who ever undergo any HPV test. Our cumulative finding that 86.4% of biopsy-confirmed CIN2+ lesions and 47.5% of HPV16-associated CIN2+ would have been detected with a urinary test alone is, in this regard, programmatically relevant: it suggests that urine could be deployed as the entry point to a triage cascade in under-screened Romanian populations, with cervical examination reserved for urine-positive women.

Beyond the virological signal, our cohort showed a steep gradient of active smoking with histological severity (24.0% in no-lesion, 36.6% in CIN1, 62.7% in CIN2+; χ^2^ = 23.71, *p* < 0.001). The CIN2+ stratum was simultaneously younger, and these two gradients should be read as interrelated rather than independent: the present analysis is unadjusted, and the diagnostic design of the study does not permit determination of which factor carries the causal weight. The smoking association reported here should therefore be interpreted as a descriptive correlate, consistent with the established literature [35], rather than as an independent predictor of high-grade disease. The magnitude of this gradient very closely mirrors the findings of Kayar et al., who in a matched case–control study of 624 high-risk HPV-positive Turkish women demonstrated that smoking—particularly when quantified by pack-years—was independently associated with progression to high-grade histopathological lesions [23]. The biological plausibility of this association has been established for decades, but its visibility in a cohort of 230 women is striking and reinforces the case for integrating smoking status into post-HPV-test risk stratification, regardless of whether the initial sample is urinary or cervical. Notably, neither the urine nor the cervical hrHPV result fully captures smoking-attributable risk, which means that urine HPV positivity in a smoker—even more than in a non-smoker—should not be dismissed as transient.

Three mechanistic considerations help frame these results. First, HPV DNA reaches the FVU stream through passive washing of exfoliated cervical and vaginal cells during voiding; consequently, the analytical signal is necessarily diluted compared with a directly sampled brushing, but it remains highly representative of the cervical reservoir because the major epithelial source is anatomically continuous [24,32]. This explains both the systematic deficit in urinary positivity (the 30 discordant cases in our data) and the very high genotype-specific concordance once a positive signal is obtained, which is what we observe for HPV16 and HPV18.

Second, the monotonic relationship between histological severity and urinary positivity is consistent with the biology of HPV persistence: high-grade lesions harbor higher integrated viral loads, more active transcription of E6/E7 oncogenes and higher epithelial turnover, all of which increase the quantity of viral DNA shed into the cervico-vaginal lumen and, downstream, into the urinary stream [23,32,33]. The genotype-specific gradient we observe—particularly for HPV16, where urinary positivity nearly quintupled across the histological strata—fits this model precisely.

Third, the role of smoking is mechanistically distinct from the role of the virus. Smoking does not increase the probability that urine will detect an existing HPV infection; rather, it modulates the probability that an existing infection progresses to a clinically significant lesion, through local immunosuppression, cotinine-mediated DNA damage and impaired clearance [23]. The combined finding of a urinary HPV signal that tracks severity and a smoking gradient that tracks the same axis therefore reflects two independent biological mechanisms converging on the same histological endpoint, and both should inform clinical management.

The principal strengths of this work are its prospective design, the inclusion of paired urinary and cervico-vaginal samples in all 230 participants, the systematic use of biopsy as the histological reference standard, the application of a contemporary multiplex genotyping assay with separate HPV16/HPV18 identification, and—distinctively—the joint reporting of agreement and diagnostic-accuracy metrics for the same cohort, with full stratification by histological grade. The use of an FVU-compatible collection workflow is supported by the contemporary VALHUDES validation literature [31] and by recent device-level analytical evaluations of the Alinity m HR HPV assay in next-generation FVU collectors [24].

A final consideration concerns the programmatic positioning of urinary HPV testing within European screening policy. On the current evidence base, urinary testing should not be proposed as an equivalent substitute for clinician-collected molecular testing within an organized, quality-assured program operating at a three-to-five-year interval. The European Guidelines and the VALHUDES framework require self-collected matrices to demonstrate non-inferior clinical sensitivity for CIN2+ on a validated assay before adoption as a primary screening modality [25], and urine has not yet cleared that threshold—a position consistent with our own relative sensitivity of 0.94 and the thirty cervical-positive/urine-negative discordant pairs. What our data do support is a targeted, complementary role: urine as a reach instrument for the never-screened and under-screened, for whom the relevant comparator is not clinician-collected testing but no testing at all. Against that comparator, a sensitivity of 86.4% for CIN2+ and a negative predictive value of 92.4% represent a substantial net gain. Urine would thus serve as the entry point of a triage cascade, with every urine-positive woman referred for clinician-collected sampling, colposcopy and biopsy as indicated—preserving, rather than displacing, the central role of the gynecologist in the diagnostic pathway.

Several limitations must be acknowledged. First, the cross-sectional design precludes inference about the longitudinal performance of urinary HPV testing, in particular about the predictive value of urinary HPV negativity over a multi-year screening interval—a question that can only be answered by cohorts with prospective re-testing such as those underway in Denmark [20] and the United Kingdom. Second, although our cohort spans the full histological spectrum, it includes by design a high proportion of women referred for abnormal cytology or prior HPV positivity, contexts in which spectrum effects elevate the prior probability of disease. Because asymptomatic screening patients represented a smaller fraction of the overall cohort, the operating characteristics we report for CIN2+ should be considered an upper bound for sensitivity and a lower bound for specificity relative to a true, unselected primary-screening setting [17,18,31]. Third, while we applied a robust extraction and preservation protocol and used the Colli-Pee device to standardize first-void urine collection, the protocol did not mandate first-morning sampling. This may have introduced minor random variation in detection efficiency. Recent evidence indicates that within-day timing has limited effect on the binary outcome of HPV detection [33], but a morning-FVU protocol may further reduce the residual cervical-positive/urine-negative discordance. Fourth, the study was conducted in three outpatient clinics in north-western Romania; replication in a fully primary-screening cohort drawn from underserved Romanian regions would strengthen external validity, particularly given the Romanian disease burden documented by Saveliev et al. [19,37] and by the recently completed Uzbek pilot in a comparable Eastern setting [26,27]. Fifth, the deliberate lack of masking for pathologists regarding the patients’ routine screening results (an ethical necessity to preserve standard-of-care treatment) may theoretically introduce a minor diagnostic review bias, although this closely reflects real-world clinical practice and was mitigated by their complete blinding to the urinary test outcomes.

Sixth, while the primary premise for deploying urinary HPV testing is its potential to overcome cultural and logistical screening barriers, we did not directly measure patient acceptability, satisfaction, or preference via structured questionnaires in our cohort. However, the superior acceptability of urine over both clinician-collected smears and vaginal self-sampling has been robustly and repeatedly established in dedicated international studies [38,39,40]. Relying on this established behavioral premise, our study was deliberately designed to focus strictly on resolving the remaining missing link: validating the diagnostic and analytical performance of this highly acceptable matrix within the specific histological spectrum of the Romanian population.

Seventh, HPV vaccination status was not recorded, and vaccinated women were not excluded. Although the near-absence of vaccination coverage in the relevant Romanian birth cohorts makes a material confounding effect improbable, we cannot formally exclude the presence of a small number of vaccinated participants, nor can we assess the influence of vaccine valency on genotype distribution, viral persistence or recurrence. Vaccination status, including valency and age at administration, will be prospectively recorded in the planned follow-up cohort.

The associations between age, active smoking and histological grade reported here are unadjusted. Because participants were recruited across three clinical scenarios of unequal pre-test disease probability, the joint distribution of these variables is conditioned on the referral pathway, and the cohort is therefore unsuited to multivariable estimation of independent risk factors for high-grade disease; the smoking gradient we describe should be read as a descriptive correlate, consistent with the established literature, rather than as an independent predictor.

Also, the genotyping assay employed resolved only HPV16 and HPV18 individually, reporting the remaining twelve high-risk genotypes as a pooled result. Although this reflects the current Romanian national screening recommendation and therefore preserves clinical realism, it prevented us from computing genotype-specific urinary–cervical concordance beyond HPV16 and HPV18, and from establishing whether the compartmental dilution effect is genotype-dependent. Given the growing recognition of HPV31, 33, 45, 52 and 58 as drivers of high-grade disease in European populations, replication using an extended-genotyping platform on the urinary matrix is a priority.

## 5. Conclusions

In this prospective cohort of 230 women spanning the full histological spectrum from no intraepithelial lesion to CIN2+, urinary HPV testing demonstrated substantial agreement with clinician-collected cervico-vaginal sampling for pooled high-risk genotypes (Cohen’s κ = 0.696), with higher concordance for HPV16 (κ = 0.755) and HPV18 (κ = 0.789). For biopsy-confirmed CIN2+, urinary testing achieved a sensitivity of 86.4% and a negative predictive value of 92.4%, closely approaching that of cervical sampling (93.7%), while its specificity was superior (56.7% vs. 43.3%). Urinary positivity rose monotonically with histological severity, from 25.0% in women without lesion to 86.4% in CIN2+, confirming that the matrix tracks clinically meaningful disease rather than transient infection.

Urinary HPV testing is therefore a clinically credible non-invasive alternative—not a full replacement—for clinician-collected sampling, and is best positioned as the entry point of a triage cascade in which cervical examination is reserved for urine-positive women. Its high negative predictive value makes it particularly suited to settings such as Romania, where the dominant obstacle is not analytical performance but low participation in conventional screening. A negative urinary result should not, however, be equated with a negative cervical result when determining the screening interval. Finally, active smoking was strongly associated with high-grade disease (62.7% in the CIN2+ stratum vs. 24.0% in women without lesion) and should be integrated into post-test risk stratification irrespective of the sampling matrix.

## Figures and Tables

**Table 1:** Demographic and clinical characteristics of the study cohort (*n* = 230).

Characteristic	*n*/Value	%/[IQR]
Total enrolled	230	100.0
Age (years)		
Mean ± SD	38.9 ± 10.4	—
Median [IQR]	36	[20,21,22,23,24,25,26,27,28,29,30]
Range	25–70	—
Smoking status		
Non-smoker	143	62.2
Smoker	87	37.8
Education		
University degree	73	31.7
Below university	157	68.3
Cervical cytology (Bethesda 2014) †		
NILM	76	33
ASC-US/LSIL	111	48.3
ASC-H/HSIL	43	18.7
Cervical histology (CIN system) ‡		
No lesion (biopsy-confirmed	10	4.3
No biopsy indications	90	39.1
CIN1	71	30.9
CIN2/CIN3/CIS	59	25.7
HPV positivity		
Cervico-vaginal hrHPV positive	151	65.7
Urinary hrHPV positive	125	54.3
Cervico-vaginal HPV16 positive	56	24.3
Urinary HPV16 positive	50	21.7
Cervico-vaginal HPV18 positive	13	5.7
Urinary HPV18 positive	12	5.2

† Bethesda System for Reporting Cervical Cytology, ‡ Within the CIN2+ stratum: 29 CIN2, 27 CIN3, 3 CIS on biopsy; 1 additional invasive squamous carcinoma was identified on the conization specimen. CIN, cervical intraepithelial neoplasia; CIS, carcinoma in situ; hrHPV, high-risk human papillomavirus.

**Table 2:** Cross-classification and Cohen’s kappa coefficients for urinary versus cervico-vaginal HPV testing.

Target	Both +	Both −	Cerv+/Urine−	Cerv−/Urine+	κ (95% CI)
Any hrHPV	121	75	30	4	0.696 (0.60–0.79)
HPV16	43	167	13	7	0.755 (0.65–0.86)
HPV18	10	215	3	2	0.789 (0.61–0.97)

Counts refer to the 230 paired observations. Confidence intervals computed from the asymptotic standard error provided by SPSS v23. All kappa coefficients were highly statistically significant (*p* < 0.001).

**Table 3:** Diagnostic performance for biopsy-confirmed CIN2+ by sampling modality (*n* = 230; 59 events).

Test	Sensitivity	Specificity	PPV	NPV
Cervico-vaginal hrHPV	91.5% (81.6–96.3)	43.3% (36.1–50.8)	35.8% (28.6–43.7)	93.7% (86.0–97.3)
Urinary hrHPV	86.4% (75.5–93.0)	56.7% (49.2–63.9)	40.8% (32.6–49.6)	92.4% (85.7–96.1)
Co-positivity (both +)	81.4% (69.6–89.3)	57.3% (49.8–64.5)	39.7% (31.4–48.6)	89.9% (82.8–94.3)

Wilson 95% confidence intervals. PPV, positive predictive value; NPV, negative predictive value.

**Table 4:** Cervico-vaginal and urinary HPV positivity stratified by histological grade.

Test	No Lesion (*n* = 100)	CIN1 (*n* = 71)	CIN2+ (*n* = 59)
Cervico-vaginal hrHPV +	34/100 (34.0%)	63/71 (88.7%)	54/59 (91.5%)
Urinary hrHPV +	25/100 (25.0%)	49/71 (69.0%)	51/59 (86.4%)
Cervico-vaginal HPV16 +	13/100 (13.0%)	19/71 (26.8%)	24/59 (40.7%)
Urinary HPV16 +	9/100 (9.0%)	13/71 (18.3%)	28/59 (47.5%)

*p* < 0.001 for the χ^2^ test across the three histological strata for each row. Linear-by-linear association also significant (*p* < 0.001), consistent with a monotone trend.

## Data Availability

The data and supporting information presented in this study are available on reasonable request from the corresponding author. The data are not publicly available due to privacy and ethical restrictions regarding patient clinical records.

## Abbreviations

The following abbreviations are used in this manuscript:

AUC	Area Under the (receiver operating characteristic) Curve
ASC-H	Atypical Squamous Cells, cannot exclude HSIL
ASC-US	Atypical Squamous Cells of Undetermined Significance
CI	Confidence Interval
CIN	Cervical Intraepithelial Neoplasia
CIN1	Cervical Intraepithelial Neoplasia grade 1
CIN2+	Cervical Intraepithelial Neoplasia grade 2 or worse
CIS	Carcinoma In Situ
DNA	Deoxyribonucleic Acid
E6/E7	HPV Oncoproteins E6 and E7
FVU	First-Void Urine
GRRAS	Guidelines for Reporting Reliability and Agreement Studies
HPV	Human Papillomavirus
HPV16	Human Papillomavirus genotype 16
HPV18	Human Papillomavirus genotype 18
hrHPV	High-Risk Human Papillomavirus
HSIL	High-Grade Squamous Intraepithelial Lesion
IQR	Interquartile Range
LSIL	Low-Grade Squamous Intraepithelial Lesion
NILM	Negative for Intraepithelial Lesion or Malignancy
NPV	Negative Predictive Value
OR	Odds Ratio
PABAK	Prevalence-Adjusted Bias-Adjusted Kappa
PBS	Phosphate-Buffered Saline
PCR	Polymerase Chain Reaction
PPV	Positive Predictive Value
ROC	Receiver Operating Characteristic
RT-PCR	Real-Time Polymerase Chain Reaction
SCCUT	South-Muntenia Cervical Cancer Screening study (multicenter study name)
SD	Standard Deviation
SPSS	Statistical Package for the Social Sciences
STARD	Standards for Reporting of Diagnostic Accuracy Studies
VALHUDES	Validation of Human Papillomavirus Assays and Collection Devices for HPV Testing on Self-Samples and Urine Samples
κ	Cohen’s Kappa Coefficient
